# Treatment Analysis in a Cancer Stem Cell Context Using a Tumor Growth Model Based on Cellular Automata

**DOI:** 10.1371/journal.pone.0132306

**Published:** 2015-07-15

**Authors:** Ángel Monteagudo, José Santos

**Affiliations:** Department of Computer Science, University of A Coruña, Campus de Elviña s/n, 15071 A Coruña, Spain; Southern Illinois University School of Medicine, UNITED STATES

## Abstract

Cancer can be viewed as an emergent behavior in terms of complex system theory and artificial life, Cellular Automata (CA) being the tool most used for studying and characterizing the emergent behavior. Different approaches with CA models were used to model cancer growth. The use of the abstract model of acquired cancer hallmarks permits the direct modeling at cellular level, where a cellular automaton defines the mitotic and apoptotic behavior of cells, and allows for an analysis of different dynamics of the cellular system depending on the presence of the different hallmarks. A CA model based on the presence of hallmarks in the cells, which includes a simulation of the behavior of Cancer Stem Cells (CSC) and their implications for the resultant growth behavior of the multicellular system, was employed. This modeling of cancer growth, in the avascular phase, was employed to analyze the effect of cancer treatments in a cancer stem cell context. The model clearly explains why, after treatment against non-stem cancer cells, the regrowth capability of CSCs generates a faster regrowth of tumor behavior, and also shows that a continuous low-intensity treatment does not favor CSC proliferation and differentiation, thereby allowing an unproblematic control of future tumor regrowth. The analysis performed indicates that, contrary to the current attempts at CSC control, trying to make CSC proliferation more difficult is an important point to consider, especially in the immediate period after a standard treatment for controlling non-stem cancer cell proliferation.

## Introduction

Cancer is a complex collection of distinct genetic diseases united by different hallmarks (traits that govern the transformation of normal cells into malignant cells). Hanahan and Weinberg [1] described these hallmarks in their 2000 article and its update in 2011 [2]. In these articles, the authors describe six essential alterations: self-sufficiency in growth signals, insensitivity to growth-inhibitory (antigrowth) signals, evasion of programmed cell death (apoptosis), limitless replicative potential, sustained angiogenesis, and tissue invasion. In the update [2], the authors included two more hallmarks: reprogramming of energy metabolism and evasion of immune destruction which emerged as critical capabilities of cancer cells. Moreover, the authors described two enabling characteristics or properties of neoplastic cells that facilitate acquisition of hallmark capabilities: genome instability and tumor-promoting inflammation (mediated by immune system cells recruited to the tumor).

All cells within the tumor compete for oxygen, reduced organic compounds and space, so cancer can be viewed, from the standpoint of the complex system theory and artificial life disciplines, as an ecological system in which cells with different mutations compete for survival. The interaction among cells generates an emergent behavior, that is, a behavior present in systems whose elements interact locally, providing a global behavior which cannot be explained by studying the behavior of a single element, but rather the group interactions [3]. Cellular Automata (CA) was the tool most employed in artificial life for studying and characterizing the emergent behavior [4] [5]. A cellular automaton is defined by a set of rules that establishes the next state of each of the sites of a grid environment given the previous state of this site and the states of its defined neighborhood, where the states can be associated with the cell states in the intended simulations of tumor growth. Thus, although computationally there are different approaches to model cancer growth and the traditional approach was to use differential equations to describe tumor growth [6], the approaches relying on cellular automata models or agent-based models facilitate modeling at cellular level, where the state of each cell is described by its local environment.

Previous works have used CA capabilities for different purposes in tumor growth modeling [7] [8] [9] [10]. However, fewer previous works have used CA models based on the presence of hallmarks. For example, Abbott et al. [11] and Spencer et al. [12] investigated the dynamics and interactions of the hallmarks in a CA model in which the main interest of the authors was to describe the likely sequences of precancerous mutations or pathways that end in cancer. They were interested in what sequences of mutations are most likely and the dependence of pathways on various parameters associated with the hallmarks. Basanta et al. [13] used a CA model based on the Hanahan and Weinberg hallmarks, and their work focuses on analyzing the effect of different environmental conditions upon the sequence of acquisition of phenotypic traits. In our previous studies, CA have been used to model the behavior of cells when hallmarks are present and in the avascular phase, with a different aim to that of previous works, given that focus was placed on the study of the multicellular system dynamics in terms of emergent behaviors that can be obtained, analyzing the relative importance of different hallmarks [14] [15] and the capability of Cancer Stem Cells (CSCs) and hallmarks to generate tumor growth and regrowth in different conditions [16] [17].

Given the interrelations among different hallmarks, in addition to the dependence of the hallmarks on some parameters, this modeling at cellular level with CA facilitates the study of the final emergent behavior, which cannot be foreseen in many cases. The objective of the present work is to analyze, by using this CA model, the effect of treatment applications on cancer growth in a cancer stem cell context. On this basis, in the next two sub-sections the cancer stem cell theory and aspects about treatments as well as related works are briefly discussed. The same methodology as in our previous work [17] is employed in order to simulate CSCs using a CA model of tumor growth based on the cancer hallmarks, and is briefly discussed in the Methods Section. This model allows for the analysis of treatment applications and, in the ‘Results’ section, the experiments performed and results obtained regarding the effect of treatments are discussed, with the presence of cancer stem cells and their regrowth capacity taken into account.

### The cancer stem cell theory

The cancer stem cell theory states that a small fraction of cancer cells is responsible for tumor growth and relapse. These CSCs have been shown to have various characteristics in common with stem cells. For example, they have the capacity to divide indefinitely [18]. Additionally, these CSCs present resistance to apoptosis and also present heterogeneity, i.e. the potential for multidirectional differentiation [19]. Consequently, departures from the classic view of cancer have recently emerged on the premise that if current treatments of cancer do not properly destroy enough CSCs, the tumor will probably reappear [20].

Several studies suggest that CSCs are relatively resistant to standard cytotoxic and radiation therapies. For example, Cortes-Dericks et al. [21] explain several factors relating to the low efficacy of cytotoxic therapies which affect rapidly proliferating cells, whereas evidence suggests that only a small fraction of CSCs actively proliferate, indicating an inherent resistance of the CSC population to this type of therapies. We will also take this into account in our modeling when a treatment against cancer cells is considered in the simulations.

### Cancer therapies

The goal of standard therapies like chemotherapy and radiotherapy is to destroy the tumor cells, while maintaining adequate amounts of healthy tissue. Optimality in treatment might be defined in a variety of ways. Some studies have been carried out in which the total amount of drug administered, or the number of tumor cells, is minimized. For example, in the models considered in [22] [23], the goal was to maximize the number of cancer cells killed by a chemotherapy agent, or to minimize the number of cancer cells at the end of the therapy session, while keeping the toxicity of the normal tissues acceptable.

In chemotherapy, the administration of one or more drugs is aimed to kill tumor cells in which the growth rate is faster than normal cells. Within this context, the mathematical modeling of anticancer chemotherapy has existed for more than four decades [24] [25]. For example, the Norton-Simon model [26] [27] states that the rate of cancer cell death in response to treatment is directly proportional to the tumor growth rate at the time of treatment. This model, for cell-cycle specific drugs, suggests that moderate early doses followed by later dose intensification would kill more tumor cells [26].

In the work of De Pillis and Radunskaya [28], the authors attempted to find equilibrium for a chemotherapy administration schedule that would kill off the tumor cells as effectively as possible, with the constraint that the treatment must not kill too many normal cells. Their optimal control algorithm dictates that a drug must be administered continuously over relatively long periods of time (on the order of days). Other authors have also applied techniques from optimal control theory to discover how chemotherapy and immunotherapy can best be combined for the effective treatment of cancer [29].

Several models predict that continuous infusion (in particular of cell-cycle phase specific drugs) is more effective than short pulses [30]. However, if the drug is applied too slowly by continuous infusion, drug resistance may develop [30]. Gardner [31] modeled this trade-off considering cell-cycle specific and cell-cycle nonspecific drugs, and used his model to provide insight into how the chance of a cure is connected with the dose and type of infusion.

These representative examples are not an exhaustive list of the works related to *in silico* studies about treatments. This present study is related to them although it will analyze the effect of treatments in a cancer stem cell context.

## Methods

### Tumor growth modeling based on cancer hallmarks

The event model used by Abbott et al. [11] in their study of the likely sequences of precancerous mutations that end in cancer was followed in order to simulate the behavior of cells when different hallmarks are acquired, as previously shown in more detail in previous works [14] [17].

In the simulation each cell resides in a site in a 3D lattice and has an artificial genome that indicates if any of five different hallmarks are activated as a consequence of mutations. The mutations occur when a cell divides: a hallmark is acquired with a hallmark mutation rate (1/*m*) defined by the parameter *m*, with default value *m* = 100000 as in [11] [12] [13]. The alterations in cell physiology that transform a normal cell into a cancer cell [1] [32] that have been considered are: *Self-Growth (SG)*, *Ignore Growth Inhibit (IGI)*, *Evasion of Apoptosis (EA)*, *Effective Immortality (EI)* and *Genetic Instability (GI)*.

Although Abbott et al. [11] considered the hallmark angiogenesis, in this study metastasis and angiogenesis are not considered, since the objectives are focused on the first avascular phases of tumorigenesis. Thus, every cell has its genome which consists of five binary hallmarks (SG, IGI, EA, EI and GI) plus two parameters particular to each cell: *telomere length* and *hallmark mutation rate*. This cell’s genome is inherited by the daughter cells when a mitotic division occurs.

In the modeling, cell mitoses are scheduled between 5 and 10 time iterations in the future for simulating the variable duration of the cell-life cycle (between 15 and 24 hours). Taking into account these time intervals, each iteration represents an average time of 2.6 hours; for example, 5000 iterations in the simulation imply an average time of 77.4 weeks.

**Fig 1:**
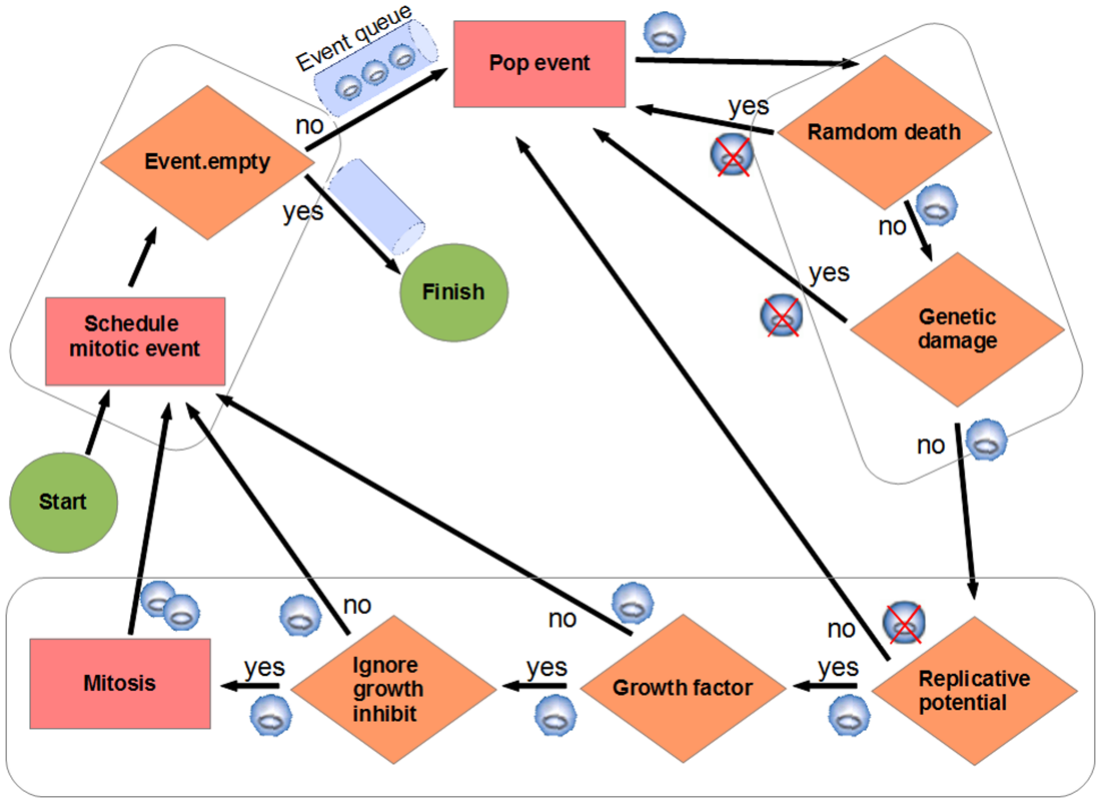
Event model used in the simulation. Mitoses are scheduled between 5 and 10 time iterations in the future. When a mitosis event is processed (popped from the event queue), several tests are performed to determine if the cell dies, continues quiescent or can perform the division. The rectangles indicate an action, a rhomb indicates a check with an associated binary question as explained in the text (Methods Section). This process is repeated to all the cells in the grid environment. Each cell is represented with a small circle. If the cell dies after a check it is represented with a crossed-circle.

The simulations begin with an initial grid full of healthy cells. Mitoses are scheduled for these cells (a mitotic event is stored, for each cell, in an event priority queue between 5 and 10 time iterations in the future). Subsequently, the simulation continuously pops the event from the event queue with the highest priority (the nearest in time), by performing the following processes (Fig 1):

1. *Random cell death test*: Cells undergo random cell death with low probability (1/*a* chance of death, with default value *a* = 1000, as in [11]).
2. *Genetic damage test*: The larger the number of hallmark mutations, the greater the probability of cell death, given by *n*/*e* (*n* is the number of hallmarks mutated, default value of *e* = 10 [11]). If “Evade apoptosis” (EA) is ON, death as a consequence of the genetic damage is not applied.
3. *Mitosis tests*:
  - *Replicative potential checking*: If the telomere length (*tl*) is 0, the cell dies, unless the hallmark “Effective immortality” (Limitless replicative potential, EI) is mutated (ON). With every cell division the telomere length is decreased in one unit, being the initial length *tl* = 50 [12].
  - *Growth factor checking*: As in [11] [12] [13], cells can perform divisions only if they are within a predefined spatial boundary, which represents a threshold in the concentration of growth factor; beyond this area (95% of the inner space in each dimension, that is, 85.7% of the 3D grid inner space) growth signals are too faint to prompt mitosis (unless hallmark SG is ON).
  - *Ignore growth inhibit checking*: If there are no empty sites in the neighborhood, the cell cannot perform a mitotic division. Following the simulation used in [11] [12], if the hallmark “Ignore growth inhibit” (IGI) is ON, then the cell competes for survival with a neighbor cell and with a likelihood of success (1/*g*, with default value *g* = 30).

If the three tests indicate possibility of mitosis:

- Increase the hallmark mutation rate if genetic instability (GI) is ON.
- Add mutations to the new cells according to the hallmark mutation rate (1/*m*).
- Decrease *tl* in both cells.
- Push events. Schedule mitotic events (push in event queue) for both cells: mother and daughter, with the random times in the future.

If mitosis cannot be applied:

- Schedule a mitotic event (in queue) for mother cell.

Thus, after the new daughter cells are created, mitosis is scheduled for each of them, and so on. Each mitotic division is carried out by copying the genetic information (the hallmark status and associated parameters) of the cell to an unoccupied adjacent space in the grid. As indicated, random errors occur in this copying process, so some hallmarks can be activated, taking into account that once a hallmark is activated in a cell, it will be never repaired by another mutation [11].

The parameters have default values which are set only as a reference. Such values, since most establish probabilities, define scenarios where particular hallmarks are more relevant in tumor progression, so we can reason about the emergent behavior in different circumstances, although it is difficult in most cases to determine a particular tumor type in which a hallmark is predominant.

### Cancer stem cell modeling

There are two models when considering the origin of cancer cells: the hierarchical model assumes that tumors are originated from CSCs that give rise to progeny with self-limited proliferative capacity where most of the cells in the tumor are genetically homogeneous. The second model is the stochastic model or clonal evolution model, which postulates that tumorigenesis is a multistep process that leads to progressively genetic alterations with the transformation of healthy cells into malignant phenotypes [33].

For the simulation of the hierarchical CSC model, the scheme that distinguishes between CSCs and Differentiated Cancer Cells (DCCs) was considered. CSCs have two defining properties: no limit in their proliferation capacity and resistance to apoptosis [19]. In this study’s model it is easy to incorporate these properties, as they imply that CSCs have acquired the hallmarks *effective immortality* (EI) and *evade apoptosis* (EA). In the simulations, a low number of CSCs in the inner area with growth factor are introduced in order to check their effect on the behavior of the multicellular system evolution.

The simulation used by Enderling and Hahnfeldt [20] regarding CSC differentiation was followed. Thus, CSCs will divide symmetrically, with a low probability (*p*_*s*_ = 0.01 in [20]) or asymmetrically (with probability 1 − *p*_*s*_) to produce a CSC and a non-stem cancer cell (Differentiated Cancer Cell—DCC). Moreover, in this simulation, in the asymmetric division, the differentiated non-stem cancer cell acquires (randomly) one of the hallmarks considered in this study’s modeling as well as the initial telomere length which defines its finite replicative potential. Therefore, this present model is more general as it considers in the CSC scheme the different capabilities of hallmarks acquired in the cells. This model does not therefore consider both models regarding the origin of cancer cells as mutually exclusive, since DCCs are genetically heterogeneous given the different hallmarks acquired in such cells. As indicated by Yap et al. [33], there is a high degree of convergence between the two models as shown, for example, in leukemia [33].

Finally, a CSC can only divide when it has available space in its immediate neighborhood. This is the same condition considered by Morton et al. [34] or Vainstein et al. [35]. For example, Morton et al. [34] state that “the probability of symmetric and asymmetric CSC division is constant and stochastic, and cells require adjacent available space to migrate or proliferate”. Similarly, in Vainstein et al. [35] the authors assume that each CSC is either “non-cycling” (quiescent) or “cycling”, i.e., the cell progresses through the cell cycle and after a fixed time period divides into two CSCs. Their non-cycling CSCs can enter the cell cycle at any time and it depends on the total cell density in the CSC’s vicinity: the more vacant (i.e., unoccupied by other cells) space available, the greater the probability that the CSC will enter the cell cycle. Nevertheless, their model of a “quorum sensing” control mechanism (direct stimulation of differentiation of a stem cell by its neighboring stem cells) was not employed, as the aforementioned model of CSC differentiation was used, taking into account a probability of symmetric and asymmetric division, as in [20] and [36].

## Results

### Simulation setup

Here the effect of a cancer treatment taking into account the presence of CSCs is simulated. Generic cancer protocols in early avascular stages of cancer are considered, given that they are ideal treatments that only kill the cancer cells, although this simplification does not change the conclusions to be drawn here. For this study representative scenarios have been considered in order to analyze the behavior of the multicellular growth when a treatment is applied.

A grid of 125000 cell sites (50 sites in each dimension), as in [11] and [12], was considered. For the simulation a number of CSCs in the inner area with growth factor (in random positions) was introduced at the beginning. The simulations begin with the grid full of cells all of which are healthy except the incorporated CSCs. Nevertheless, the emergent growth behaviors are independent of this initial condition (the initial number of healthy cells) as well as the grid size, as indicated in previous works [14] [17].

As previously explained, CSCs will divide symmetrically, with the same probability used by Enderling and Hahnfeldt [20] (*p*_*s*_ = 0.01), or asymmetrically to produce a CSC and a non-stem cancer cell with probability 1 − *p*_*s*_. Regarding the number of CSCs introduced from the beginning, this corresponds to a small percentage of the grid size (1–5%) since the proportion of identified CSCs varies among different cancers. For example, Ricci-Vitiani et al. [37] showed that tumorigenic cells in colon cancer population (with CD133+ marker) accounts for about 2.5% of the tumor cells and Korkaya et al. [38] determined that CSCs comprise 1–5% of primary tumors in human mammary carcinomas.

### Effect of treatments on regrowth behavior

In order to explain the effect of treatments in a CSC context, a scenario with high invasion potential where the value of parameter *g* was set to 5 and the rest of the hallmark parameters were set to their standard values in the runs was considered. Nevertheless, the explanations and conclusions would be similar in other scenarios. This therefore corresponds to a scenario with a high invasion potential by ignoring the contact inhibition mechanism (with probability 1/*g*), since such a low value in parameter *g* (5) was used. The parameters set in this scenario are the same used by Spencer et al. [12] in their case to study how the sequence of acquired mutations affects the timing and cellular makeup of the resulting tumor. Thus, cells that have acquired the hallmark *ignore growth inhibit* (IGI) will have a high ability to invade the surrounding space. Cervical cancer is an example, whereas, on the contrary, the naked mole-rat displays hypersensitivity to contact inhibition which confers upon it an extraordinary resistance to cancer [39].

**Fig 2:**
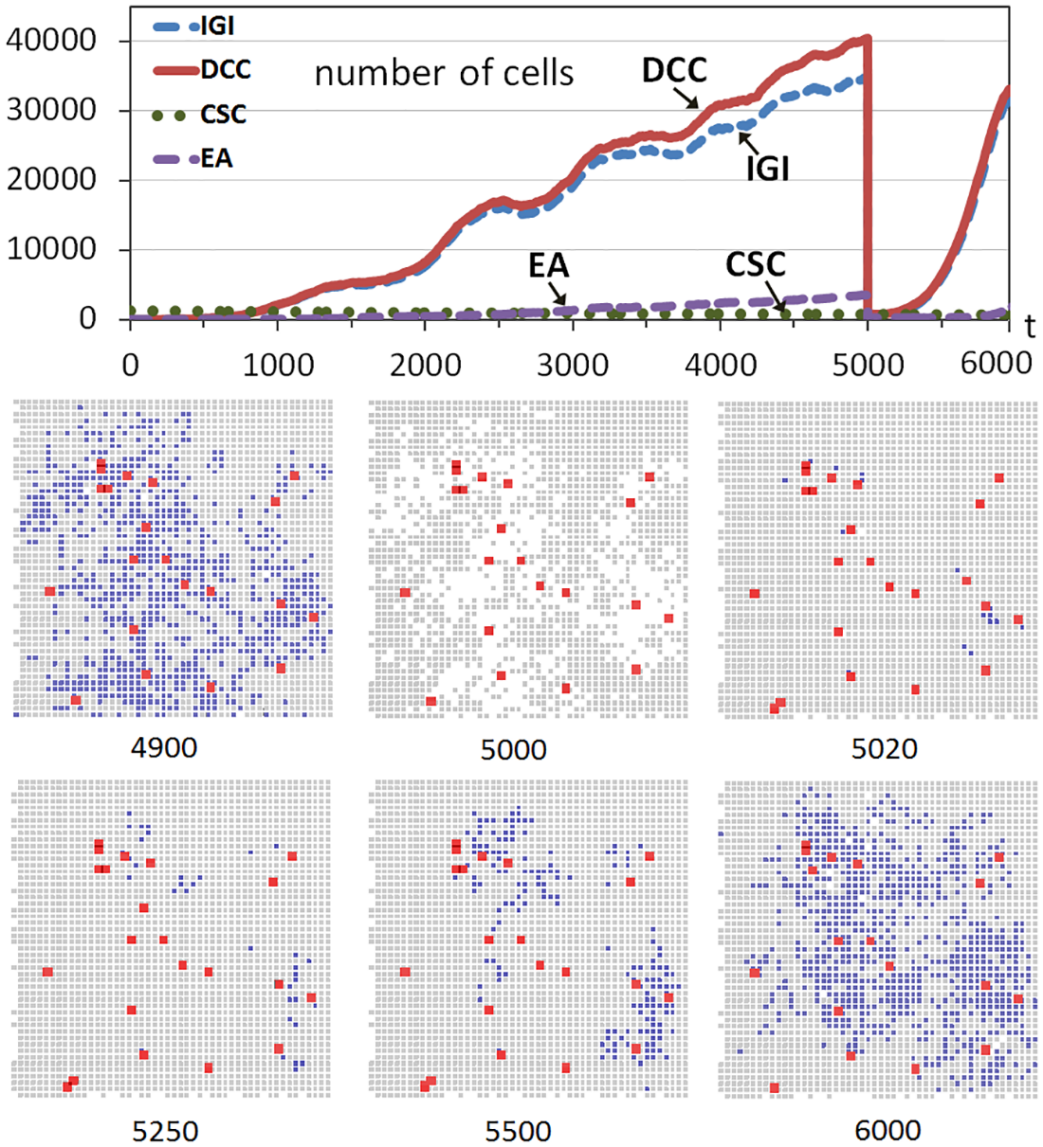
Tumor regrowth after a high-intensity treatment. The graph shows the evolution through time iterations of the number of Differentiated Cancer Cells—DCCs (continuous red line) with g = 5 while the rest of the parameters were set to their standard values. A number of Cancer Stem Cells (CSCs) corresponding to 1% of the grid size is introduced from the beginning of the simulation (dashed green line). At iteration 5000 the 100% of DCCs is killed. The graph also shows the evolution of the two most predominant hallmarks acquired in DCCs (IGI and EA). The bottom part shows snapshots of central sections of the multicellular system evolution corresponding to different time iterations (Colors: Gray—healthy cells, Blue—DCCs, Red-enlarged size—CSCs).

Fig 2 includes an example of this scenario with a run of the system evolution. The upper part of Fig 2 shows the evolution through time iterations of non-stem cancer cells (DCCs) and CSCs when the number of CSCs corresponds to 1% of the grid size (125000), CSCs that were introduced at the beginning in random positions of the inner area with growth factor. As this study has explained, the grid is initially full of cells which are practically all healthy except for the CSCs incorporated. Fig 2 also shows the evolution of the two most predominant hallmarks (considering the hallmarks acquired in all DCCs) during the multicellular system evolution.

Since there is not practically any free site, most CSCs remain quiescent until they have free space to perform mitoses. Given the low probability of symmetric division the number of CSCs remains stable during the 6000 iterations shown in Fig 2. However, the non-stem cancer cells begin to grow due to the asymmetric division of CSCs and because of the acquisition of the hallmark *ignore growth inhibit* (IGI) in few of such DCCs, which can rapidly proliferate because of their advantage in this situation with practically no free sites (the daughters acquire the same hallmark). As more mutations appear in this proliferation of DCCs, the apoptotic process begins to be an important limit for their proliferation. Therefore, as an evolutionary and emergent consequence, the hallmark *evade apoptosis* (EA) also appears in many DCCs to avoid the apoptotic process. The fluctuations in the expansion of DCCs are because most cancer cells have the hallmark IGI acquired. However, when the concentrations or clusters of cells with IGI acquired reach a certain size, then the apoptotic process can decrease their size for a small time, until reaching another lower size where the proliferation continues again.

At time iteration 5000 the 100% of non-stem cancer cells is killed, simulating a (perfect) cancer therapy and causing the drastic drop of the non-stem cancer cells. Nevertheless, since CSCs are more resistant to therapeutic interventions, such as chemotherapy or irradiation, compared with their differentiated counterparts [36], the CSCs remain in the simulation.

The bottom part of Fig 2 shows cross-sections of this multicellular system evolution at different time iterations. These cross-sections correspond to 2D sections of a plane that crosses the center of the grid, which show the expansion of the tumor cells (DCCs), with the colonization of many areas previously started with some DCCs as consequence of the continuous presence of CSCs (shown with enlarged sizes in the cross-sections). The snapshot at *t* = 5000 shows how these DCCs are eliminated by the effect of treatment. The next snapshots show how the healthy cells that have not performed the maximum number of divisions fill the space rapidly (see for example the cross-sections at t = 5020 and 5250), but the non-stem cancer cells recover quickly because the CSCs not killed produce non-stem cancer cells again. These DCCs produce an evolution pattern similar to the one at the beginning of the simulation, as shown in the cross-sections and in the upper part of Fig 2.

Nevertheless, there is an important and evident difference with respect to the first growth pattern of DCCs: this second proliferation on non-stem cancer cells is faster. This is because after the elimination of DCCs, CSCs had more opportunities to proliferate and differentiate in the small amount of time until the grid is completely filled with mostly healthy cells. Therefore the few non-stem cancer cells, after the differentiation, can produce the faster proliferation of non-stem cancer cells. On the other hand, in the first part of the simulation, many iterations were needed to obtain a few DCCs with the hallmark IGI acquired, given the few possibilities of cell division and the low hallmark mutation rate.

**Fig 3:**
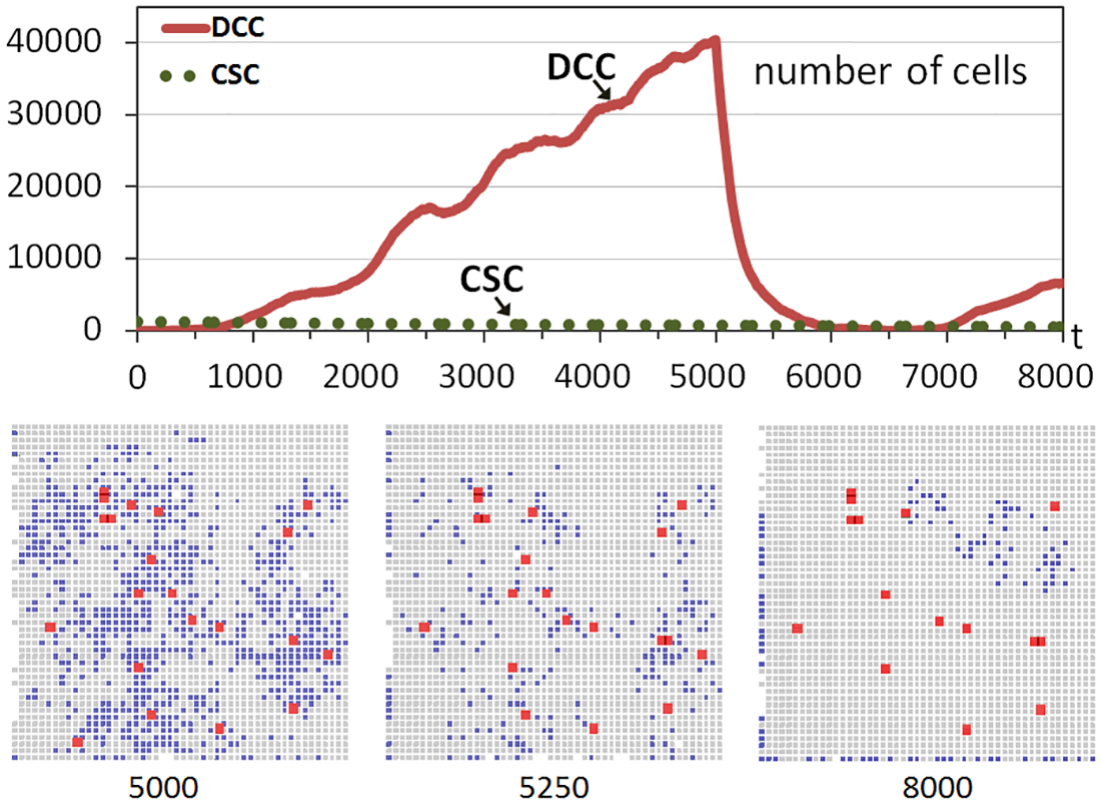
Tumor regrowth after a low-intensity treatment. The graph shows the evolution through time iterations of the number of Differentiated Cancer Cells—DCCs (continuous red line) with g = 5 while the rest of the parameters were set to their standard values. A number of Cancer Stem Cells (CSCs) corresponding to 1% of the grid size is introduced from the beginning of the simulation (dashed green line). Between iterations 5000 and 6000 DCCs are killed with a probability of 1%. The bottom part shows snapshots of central sections of the multicellular system evolution corresponding to three different time iterations (Colors: Gray—healthy cells, Blue—DCCs, Red-enlarged size—CSCs).

However, the situation would change if CSCs did not have the opportunity to proliferate and differentiate in the short interval with sufficient available free space after the treatment. The experiment was repeated but this time by considering a situation where the treatment kills DCCs gradually and within a given period. Fig 3 shows another run although now the treatment is applied between iterations 5000 and 6000, killing 1% of DCCs in each iteration. Now there is an important difference after the beginning of the treatment, because in the next iterations the healthy cells rapidly replace the free sites that are appearing as a consequence of the elimination of a limited number of DCCs. Most of the few CSCs are therefore immediately surrounded by cells, without the possibility of proliferation and differentiation as in the previous case. This can be observed in the snapshots corresponding to iterations 5000 and 5250.

At time iteration *t* = 6000 the treatment is ceased and DCCs begin to proliferate again. Nevertheless, the slope in the regrowth is not as high as in the previous case (Fig 2) because the treatment strategy did not allow the differentiation of a significant number of CSCs as in that first case. This can be seen in the evolution graphs (Figs 2 and 3) or in the snapshots. For example, the comparison of the snapshot of Fig 2 at *t* = 6000 (1000 iterations after the beginning of the regrowth) and the snapshot at *t* = 8000 of Fig 3 (2000 iterations after the beginning of the regrowth since the treatment ceases at *t* = 6000), clearly shows the faster increase of DCCs using a high intensity treatment in a short period. This therefore indicates and explains that this strategy, the application of a treatment against non-stem cancer cells with low intensity and with a longer period, is better regarding the future regrowth of the tumor behavior. It should be noted that this conclusion is a consequence of the higher probability of CSCs proliferating and differentiating which is favored with treatments with high intensity. Moreover, this effect is independent of the particular advantage of DCCs proliferating, although in this example such a scenario, where cells with the hallmark IGI acquired are the most predominant, was chosen.

This aspect in the avascular phase was analyzed. However, it should be further noted that the analysis will be analogous in a vascular phase or when CSCs evade the main tumor. The inclusion of metastasis and motility for CSCs could provide insights about other aspects, such as possible morphologies of the growing tumor, as studied, for example, in the work of Sottoriva et al. [36] in order to analyze the different infiltrative morphologies beyond the borders of the main tumor mass.

### Treatment scheduling

The objective of this part of the study is to analyze treatment scheduling in a CSC context by taking into account the effects of treatment intensity and application period in future tumor regrowth. Two representative scenarios were considered: the one previously used where the predominant hallmark is *ignore growth inhibit*, and another scenario dominated by the hallmark *evasion of apoptosis* when using an increased mutation rate.

**Fig 4:**
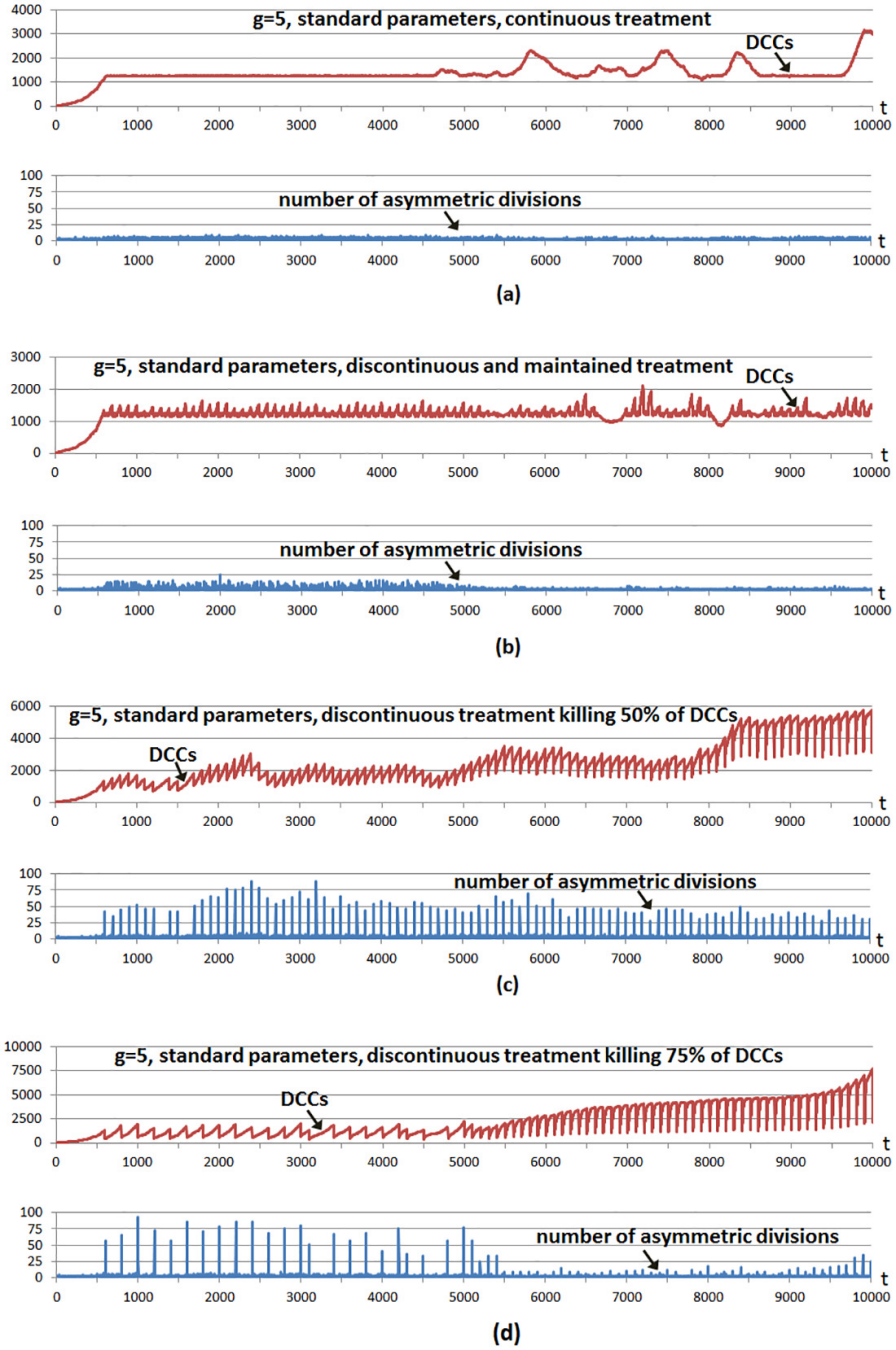
Different treatment strategies in a high invasion potential scenario. The graphs show the evolution through time iterations of the number Differentiated Cancer Cells—DCCs (continuous red line) with g = 5 while the rest of the parameters were set to their standard values. A number of Cancer Stem Cells (CSCs) corresponding to 5% of the grid size is introduced from the beginning of the simulation (not shown). In all cases the treatment is applied from the beginning only if the number of DCCs is equal or over a threshold value (1% of the grid size). a) A treatment is applied continuously, killing 1% of DCCs. b) The treatment is applied every 100 time iterations, continuously killing 10% of DCCs during 60 iterations. c) The treatment is applied every 100 time iterations, killing 50% of DCCs. d) The treatment is applied every 100 time iterations, killing 75% of DCCs. In the four cases the bottom parts show the number of asymmetric divisions in CSCs.

Fig 4 shows a multicellular system evolution with the same hallmark parameters used in Fig 2; that is, using a high invasion potential scenario in which the parameter *g* was set to 5 and the rest of the hallmark parameters were set to their standard values. The number of incorporated CSCs is 5% of the grid size, a larger number with respect to previous cases to show better CSC implications. A treatment is started from the beginning, and considering the four cases shown in Fig 4. In all cases the treatment is applied using a simple control: it is administered unless the number of DCCs is under a threshold value corresponding to 1% of the grid size. We use this so we can infer conclusions about tumor regrowth without considering the extreme case where the number of DCCs is reduced to zero, as in previous explanatory examples.

In the first case (Fig 4a) the treatment kills 1% of DCCs in each iteration. In the second case (Fig 4b) the treatment kills 10% of DCCs in each iteration, maintaining the treatment during 60 iterations (6.5 days) and repeating the strategy periodically every 100 iterations (10.8 days). In the third case (Fig 4c) the treatment is applied every 100 time iterations although now 50% of DCCs in such iterations are killed. The fourth case (Fig 4d) is similar although 75% of DCCs are killed when the treatment is applied every 100 time iterations. The bottom subfigures in the four cases show the number of CSC asymmetric divisions in every simulation iteration.

In the first strategy (Fig 4a), the simple adaptive mechanism of treatment application maintains very close the number of DCCs to the threshold value, with very few CSC asymmetric divisions. The number of DCCs occasionally presents small increases and oscillations. The fluctuations in the expansion of DCCs are now because some cancer cells have acquired the hallmark *self-growth* (SG) in the outer part of the grid. In this area there is not growth factor, so empty sites appear quickly since only cells with SG acquired can perform mitosis. When a DCC with SG acquired appears in the outer area, a fast increase of DCCs occurs which is not controlled with the treatment. When these DCCs have colonized the outer area, other factors reduce their number, being the main reason that some cells have also acquired the hallmark *ignore growth inhibit* (IGI), given such rapid proliferation. Therefore the same reason for the oscillations in the number of DCCs in previous Figures appears again.

In the third case (Fig 4c), after 50% of DCCs have been killed, these recover again owing to the fast proliferation of the remaining DCCs and to the CSC proliferation and differentiation which is now favored by the fast DCC elimination. This last contribution has a higher importance when the number of remaining DCCs is lower. The run also indicates that it is not necessary to kill 100% of DCCs in order to show the fast increase of DCCs as a consequence of the greater CSC differentiation possibility. The fourth case is similar (Fig 4d), when the treatment kills 75% of DCCs in particular iterations, although in this case the treatment is applied fewer times with respect to the third case, since the threshold is reached in less occasions. As in the previous case, killing a considerable number of DCCs favors CSC differentiation, therefore DCCs recover again to obtain a larger number of DCCs. The second case (Fig 4b) is a mixture of both strategies, as it uses a lower intensity with respect to the last cases but it is maintained during a certain number of iterations.

It should be noted that after time iteration 5000, and since most DCCs are located in the outer area (cells with hallmark *self-growth*), when the treatment is applied it kills cells mostly in this area. The empty cells are therefore mostly located in that area and, consequently, only the few CSCs located there can perform the asymmetric divisions. This explains why there is a lower number of asymmetric divisions after time iteration 5000. This effect is clearer when many DCCs are killed, as when the treatment is applied every 100 time iterations killing 75% of DCCs (Fig 4d) and when the treatment is applied every 100 time iterations, continuously killing 10% of DCCs during 60 iterations (Fig 4b), whereas the effect is less clear when it is applied every 100 time iterations killing 50% of DCCs (Fig 4c).

**Fig 5:**
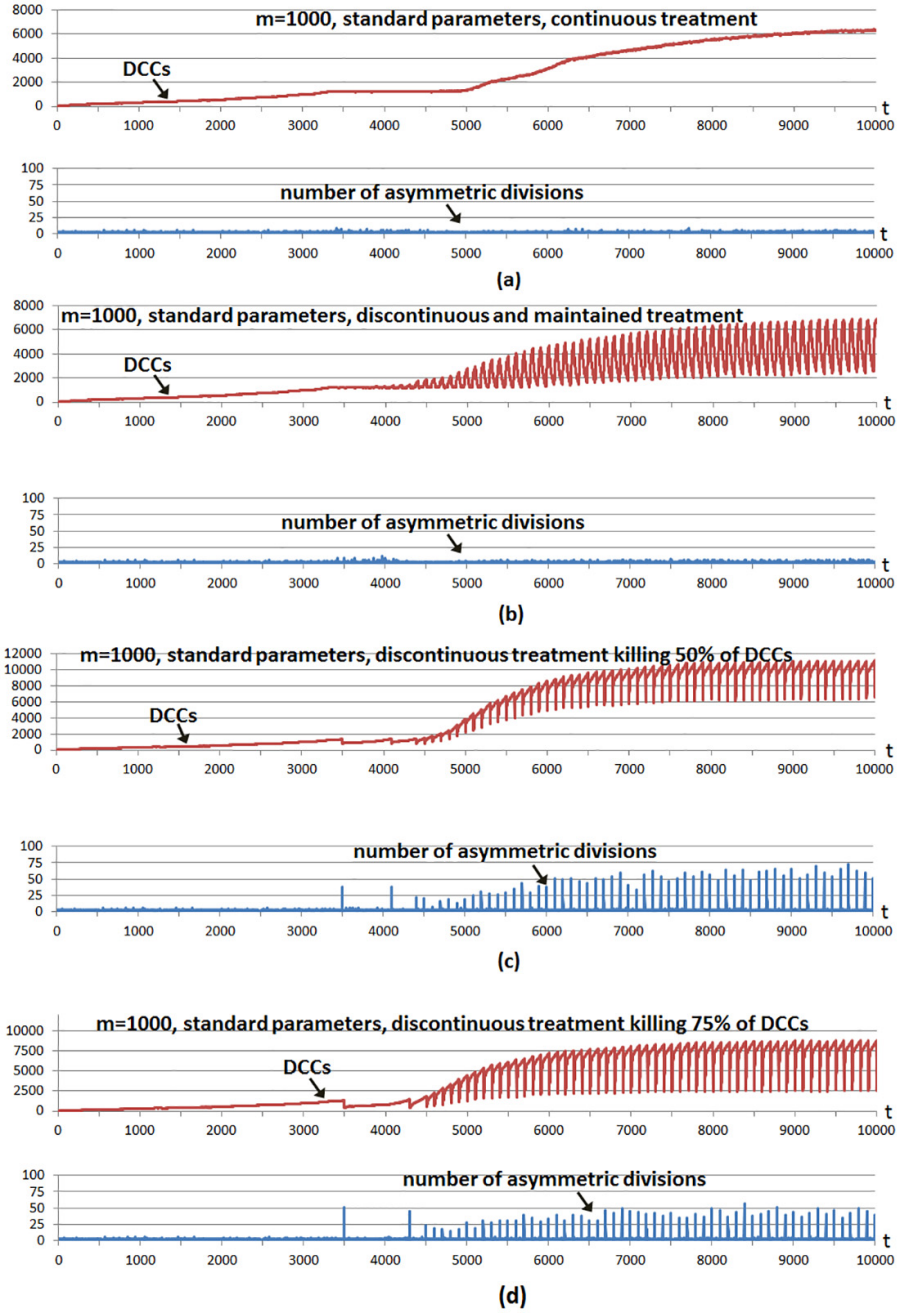
Different treatment strategies in a high hallmark mutation scenario. The graphs show the evolution through time iterations of the number Differentiated Cancer Cells—DCCs (continuous red line) with m = 1000 while the rest of the parameters were set to their standard values. Same setup as in Fig 4: a number of CSCs corresponding to 5% of the grid size is introduced from the beginning. A treatment is applied from the beginning only if the number of DCCs is equal or over a threshold value (1% of the grid size). a) A treatment is applied continuously, killing 5% of DCCs. b) The treatment is applied every 100 time iterations, continuously killing 10% of DCCs during 60 iterations. c) The treatment is applied every 100 time iterations, killing 50% of DCCs. d) The treatment is applied every 100 time iterations, killing 75% of DCCs.

The experiment was repeated with standard parameters and a higher hallmark mutation rate (*m* = 1000) with respect to the standard value (*m* = 100000). The parameter *m* sets the time scale of the simulation as it determines how fast tumor growth behavior can appear, although the onset of tumor growth depends on the parameter values of other hallmarks. Given the increased hallmark mutation rate, the hallmark *evade apoptosis* (EA) is the most predominant in DCCs in evading the apoptotic process. Fig 5 shows the same cases considered in the previous example, except that in this case the treatment continuously applied (Fig 5a) kills 5% of DCCs in each iteration. This higher killing frequency (with respect to the previous example) is because it is necessary to decrease the number of DCCs faster given the higher hallmark mutation rate. In all cases the number of incorporated CSCs is again 5% of the grid size. The cases considered in Fig 5 show once more that the same conclusions can be inferred, given that with discontinuous treatments more number of asymmetric divisions are obtained, whereas with continuous treatment, such a number of asymmetric divisions is near zero and consequently fast regrowth of DCCs is not favored. This is also the case with a low intensity treatment maintained during an appropriate period (Fig 5b) in order to decrease the number of DCCs at the same time that low intensity does not favor the proliferation and differentiation of CSCs.

## Discussion

As Enderling et al. state [40] “Mathematical modeling of biological systems has proven to be a useful tool in understanding complex dynamics and identifying the contribution of different mechanisms and parameters in the evolving population. In recent years the number of mathematical models for tumor growth, avascular and vascular, has increased dramatically and a straightforward application of such models has been to simulate treatment response and predict novel strategies”.

In this case of tumor growth modeling, as indicated by Savage [41] “Researchers have tended to focus on genes and proteins, but to understand and fight the disease, it must be viewed as a system, rather than merely as a set of cellular activities”. Thus, “the recent focus on genetics and pathways in individual cells has caused many researchers to neglect the systemic view” [41]. Bearing in mind these considerations, CA approaches facilitate the modeling at cellular level, where the state of each cell is described by its local environment, as it was commented in the Introduction. Along this line we used a CA model based on the presence of the Hanahan and Weinberg hallmarks in the cells, which is useful to study the emergent behavior in scenarios related with the different relevance of hallmarks.

Different strategies when a treatment is applied were analyzed by taking into account the implications of CSC presence. These cells are resistant to usual present therapies, so these are the main cause responsible for tumor regrowth after treatments. As stated by Sottoriva et al. [36] “Therapy which fails to target the CSC population is not only unsuccessful in curing the patient, but also promotes malignant features in the recurring tumor. These include rapid expansion, increased invasion, and enhanced heterogeneity”. This present analysis is therefore different to the studies focused on treatment applications to control non-stem cancer cells, given that its aims center upon future tumor regrowth in the long term as a consequence of the CSC behavior.

Vainstein et al. [35] employed a CSC simulation that takes into account a quorum sensing model (higher CSC density in the microenvironment promotes CSC differentiation). Their model considers “cycling CSCs” which are those CSCs that progress through the cell cycle and after a fixed time period divide into two CSCs, whereas the “non-cycling” CSCs are the quiescent CSCs that enter the cell cycle depending on the total cell density in the CSC’s vicinity. Even the authors remark that their model’s assumptions differ from those of other models of CSC dynamics, which either do not consider differentiation as a process triggered by environmental feedback or do not take into account the influence of neighboring cells on the differentiation decision (such as the model of Enderling and Hahnfeldt [20] and this study’s model), they also showed that “accelerated death of DCCs (represented in their model by limited lifespan) decreased the number of DCCs, but increased the number of cycling CSCs”. Although using a different model of CSC differentiation, this aspect of their model is similar to the results of this study regarding the relation between DCC elimination and CSC differentiation. Moreover, those authors [35] stated that neither stimulation of CSC differentiation or inhibition of CSC proliferation alone is sufficient for complete CSC elimination and cancer cure, since each of these two therapies affects a different subpopulation of CSCs. A “differentiation therapy” (like retinoic acids and drugs targeting tumor epigenetic changes) forces CSCs to differentiate terminally and lose their self-renewal property [42]. Vainstein et al. [35] therefore proposed that, in clinical trials, CSC differentiation therapy should only be examined in combination with chemotherapy to substantially reduce the population sizes and densities of all types of cancer cells.

Our results concerning the faster regrowth after a treatment are also in agreement with the results of Hillen et al. [43] when they comment regarding “tumor growth paradox”. The authors define this paradox as an “accelerated tumor growth with increased cell death as, for example, can result from the immune response or from cytotoxic treatments”. They showed that if DCCs compete with CSCs for space and resources, the first cells can prevent CSC division and drive tumors into dormancy. Conversely, if this competition is reduced by death of DCCs, as the authors state “the result is a liberation of CSCs and their renewed proliferation, which ultimately results in larger tumor growth”. Their model only considers DCCs with limited proliferation capability, whereas the model employed in this study considers the different hallmarks that a DCC can acquire; therefore the different capabilities of the individual hallmarks can be analyzed in different situations. Nevertheless, the conclusions drawn here are the same in different scenarios characterized by the relative importance of the different hallmarks and clearly indicate that the treatments applied must not promote CSC division.

Finally, the effects of different treatment strategies were analyzed by using continuous low-intensity treatments and periodic high-intensity treatments. The former presents an advantage as a continuous low-intensity treatment which does not favor CSC proliferation and differentiation, and therefore allows easy control of future tumor regrowth. On the contrary, the latter possibility of discontinuous periodic treatments with high intensity levels favors CSC proliferation and differentiation in the short term, and consequently favors future tumor regrowth. Thus, this analysis indicates that making CSC proliferation more difficult is an important point to consider, especially in the immediate period after a standard treatment for controlling non-stem cancer cell proliferation. As stated by Han et al. [42] “maintaining the cells in a quiescent state by blocking specific receptors and signaling pathways within the CSC niche can inhibit CSC functions of tumor initiation and metastasis”. Nevertheless, the current attempts at CSC control are focused on alternatives for CSC elimination and induction of CSC differentiation [42] [44].
